# A machine learning model to predict neurological deterioration after mild traumatic brain injury in older adults

**DOI:** 10.3389/fneur.2024.1502153

**Published:** 2025-01-03

**Authors:** Daisu Abe, Motoki Inaji, Takeshi Hase, Eiichi Suehiro, Naoto Shiomi, Hiroshi Yatsushige, Shin Hirota, Shu Hasegawa, Hiroshi Karibe, Akihiro Miyata, Kenya Kawakita, Kohei Haji, Hideo Aihara, Shoji Yokobori, Takeshi Maeda, Takahiro Onuki, Kotaro Oshio, Nobukazu Komoribayashi, Michiyasu Suzuki, Taketoshi Maehara

**Affiliations:** ^1^Department of Neurosurgery, Tokyo Medical and Dental University, Bunkyo-ku, Japan; ^2^Institute of Education, Innovative Human Resource Development Division, Tokyo Medical and Dental University, Bunkyo-ku, Japan; ^3^Department of Neurosurgery, School of Medicine, International University of Health and Welfare, Narita, Japan; ^4^Emergency Medical Care Center, Saiseikai Shiga Hospital, Ritto, Shiga, Japan; ^5^Department of Neurosurgery, NHO Disaster Medical Center, Tachikawa, Japan; ^6^Department of Neurosurgery, Tsuchiura Kyodo General Hospital, Tsuchiura, Ibaraki, Japan; ^7^Department of Neurosurgery, Kumamoto Red Cross Hospital, Kumamoto, Japan; ^8^Department of Neurosurgery, Sendai City Hospital, Sendai, Miyagi, Japan; ^9^Department of Neurosurgery, Chiba Emergency Medical Center, Chiba, Japan; ^10^Emergency Medical Center, Kagawa University Hospital, Kita-gun, Kagawa, Japan; ^11^Department of Neurosurgery, Yamaguchi University School of Medicine, Ube, Yamaguchi, Japan; ^12^Department of Neurosurgery, Hyogo Prefectural Kakogawa Medical Center, Kakogawa, Hyogo, Japan; ^13^Department of Emergency and Critical Care Medicine, Graduate School of Medicine, Nippon Medical School, Bunkyo-ku, Japan; ^14^Department of Neurological Surgery, Nihon University School of Medicine, Itabashi-ku, Japan; ^15^Department of Emergency Medicine, Teikyo University School of Medicine, Itabashi-ku, Japan; ^16^Department of Neurosurgery, St. Marianna University School of Medicine, Kawasaki, Kanagawa, Japan; ^17^Iwate Prefectural Advanced Critical Care and Emergency Center, Iwate Medical University, Yahaba, Iwate, Japan

**Keywords:** mild traumatic brain injury, neurological deterioration, machine learning, predictive model, XGBoost

## Abstract

**Objective:**

Neurological deterioration after mild traumatic brain injury (TBI) has been recognized as a poor prognostic factor. Early detection of neurological deterioration would allow appropriate monitoring and timely therapeutic interventions to improve patient outcomes. In this study, we developed a machine learning model to predict the occurrence of neurological deterioration after mild TBI using information obtained on admission.

**Methods:**

This was a retrospective cohort study of data from the Think FAST registry, a multicenter prospective observational study of elderly TBI patients in Japan. Patients with an admission Glasgow Coma Scale (GCS) score of 12 or below or who underwent surgical treatment immediately upon admission were excluded. Neurological deterioration was defined as a decrease of 2 or more points from a GCS score of 13 or more within 24 h of hospital admission. The model predictive accuracy was judged with the area under the receiver operating characteristic curve (AUROC) and the area under the precision-recall curve (AUPRC), and the Youden index was used to determine the cutoff value.

**Results:**

A total of 421 of 721 patients registered in the Think FAST registry between December 2019 and May 2021 were included in our study, among whom 25 demonstrated neurological deterioration. Among several machine learning algorithms, eXtreme Gradient Boosting (XGBoost) demonstrated the highest predictive accuracy in cross-validation, with an AUROC of 0.81 (±0.07) and an AUPRC of 0.33 (±0.08). Through SHapley Additive exPlanations (SHAP) analysis, five important features (D-dimer, fibrinogen, acute subdural hematoma thickness, cerebral contusion size, and systolic blood pressure) were identified and used to construct a better performing model (cross-validation AUROC of 0.84 and AUPRC of 0.34; testing data AUROC of 0.77 and AUPRC of 0.19). At the cutoff value from the Youden index, the model showed a sensitivity, specificity, and positive predictive value of 60, 96, and 38%, respectively. When neurosurgeons attempted to predict neurological deterioration using the same testing data, their values were 20, 94, and 19%, respectively.

**Conclusion:**

In this study, our predictive model showed an acceptable performance in detecting neurological deterioration after mild TBI. Further validation through prospective studies is necessary to confirm these results.

## Introduction

With population aging, the number of elderly patients with traumatic brain injury (TBI) continues to increase worldwide (1, 2). Although the causes of head injury among elderly patients with TBI, such as falls, are considered minor in this population, they are known to result in worse life and functional outcomes in elderly patients than in younger patients (3–6). One factor contributing to the poor prognosis of TBI in elderly patients is the increased incidence of neurological deterioration, which refers to the progression from an initial mild state—where patients can talk and communicate—to a state where consciousness impairment advances within a short period. Neurological deterioration has been recognized as a poor prognostic indicator in the management of head trauma. In particular, elderly patients are thought to be prone to neurological deterioration due to age-related brain atrophy, which may obscure the manifestations of intracranial hemorrhage or brain swelling in the early stages of injury, leading to underestimation of the initial severity of head trauma (7). Predicting the progression to neurological deterioration and subsequently initiating appropriate monitoring and interventions before the occurrence of deterioration may lead to improved outcomes for elderly patients with TBI.

Factors that have been reported to affect neurological deterioration include the presence of acute subdural hematoma, the use of anticoagulant medications (8), and elevated D-dimer levels on admission. However, there is no established way to assess the risk of neurological deterioration in each patient, possibly because the occurrence of neurological deterioration is relatively rare, so it is difficult to extract data on multiple independent factors related to this condition and construct predictive models via conventional statistical methods.

In recent years, many studies have demonstrated that machine learning enables the development of more accurate predictive models than traditional statistical methods do, such as in predicting the length of stay in the ICU for trauma patients or the risk of developing epilepsy after TBI. Although one of the weaknesses in utilizing machine learning analysis lies in its black-box nature, which possibly hinders its widespread use owing to the lack of clinical interpretability, recent advancements in interpreting the decisions made by machine learning models are expected to facilitate the application of machine learning in clinical settings (9).

In this study, we developed a predictive model using machine learning algorithms to predict the occurrence of neurological deterioration in elderly patients with mild TBI. Furthermore, we examined the clinical validity of the constructed predictive model by using the SHapley Additive exPlanations (SHAP) (10) explainable artificial intelligence (XAI) method.

## Methods

### Study population

This study was approved by the Medical Research Ethics Committee of Tokyo Medical and Dental University (M2019-210) and all the participating institutions. The requirement for informed consent was waived because of the observational nature of this study.

In this study, analysis was conducted using data from the Think FAST registry (11), a multicenter prospective database that contains data on hospitalized patients aged 65 years and older with head injuries. The participating institutions include Iwate Medical University, Sendai City Hospital, Tsuchiura Kyodo General Hospital, Chiba Emergency Medical Center, Teikyo University Hospital, Nippon Medical School Hospital, Nihon University Hospital, National Disaster Medical Center, St. Marianna University Hospital, Tokyo Medical and Dental University Hospital, Saiseikai Shiga Hospital, Hyogo Prefectural Kakogawa Medical Center, Kagawa University Hospital, Yamaguchi University Hospital, and the Japanese Red Cross Kumamoto Hospital. Patient registration was conducted between December 2019 and May 2021, resulting in the inclusion of data from 721 patients. This study followed the Strengthening the Reporting of Observational Studies in Epidemiology (STROBE) reporting guidelines (12).

### Definition of neurological deterioration

In this study, neurological deterioration was defined as a decrease of 2 or more points in the Glasgow Coma Scale (GCS) score in patients whose admission GCS score was 13 points or higher and who were treated with conservative management on admission.

### Features

The Think Fast registry contains the prospectively registered data of hospitalized patients aged 65 years and older with head trauma. In this study, the following data were extracted from the database: age, sex, vital signs (systolic blood pressure, heart rate), consciousness level (evaluated via the GCS), laboratory data (platelet count, prothrombin time-international normalized ratio (PT-INR), activated partial thromboplastin time (APTT), D-dimer level, fibrinogen level), head computed tomography (CT) findings [acute subdural hematoma thickness (mm), acute epidural hematoma thickness (mm), cerebral contusion diameter (mm), presence of traumatic subarachnoid hemorrhage, skull vault fracture, skull base fracture, midline shift (mm), appearance of basal cisterns (normal/compressed/disappeared)], mechanism of injury (traffic accident/fall), time from injury to hospital arrival (minutes), administration of hemostatic agents (tranexamic acid, carbazochrome), antithrombotic drug intake (number of antiplatelet drugs or anticoagulants), and reversal therapy (vitamin K, fresh frozen plasma, four-factor prothrombin complex concentrate, idarucizumab, platelet transfusion). Vital signs, consciousness levels, and laboratory data were collected at the time of the patient’s arrival at the emergency room. CT findings were also based on examinations performed at the time of arrival, and the measurements were calculated from the images of each patient.

### Machine learning algorithms

Prediction models, including logistic regression (13), support vector machine (with linear and radial basis function (RBF) kernels) (14), eXtreme Gradient Boosting (XGBoost) (15, 16), and random forest models (17, 18), were constructed via machine learning algorithms.

The predictive accuracy of the models was compared through cross-validation. The best performing algorithm was then selected, and SHAP (10) values were computed to visualize their relative importance for each feature. Furthermore, the dimensionality of the feature space was reduced according to the importance values, and the resulting features were used to create the most accurate prediction model.

Python version 3.9 was used to build the machine learning models and analyze missing values and SHAP values. Several Python modules were employed for this task, including numpy 1.23.2, scikit-learn 1.4.0, matplotlib-base 3.6.3, pandas 1.5.3, XGBoost 1.7.1, pyampute 0.0.3, and SHAP 0.41.0.

### Preprocessing

Supplementary Figure 1 illustrates the percentage of missing values for each feature in the whole dataset. In particular, the proportion of missing values related to the coagulation system was greater than that related to other systems. Little’s missing completely at random (MCAR) test yielded a *p* value of 0.93, indicating that the missing values were not MCAR. Therefore, we adopted four approaches to handle the missing data in this study: (1) k-nearest neighbors (19); (2) multiple imputation (20); (3) random forest regression (21); and (4) no imputation, employed only in the XGBoost model since only XGBoost can handle data containing missing values.

The entire dataset was randomly divided at a 3:2 ratio into training and validation datasets (60% of the data), which were used for model creation with threefold cross-validation (*k* = 3), and a testing dataset (the remaining 40% of the data), which was used to test the performance of the model. During cross-validation, hyperparameter tuning within the ranges (shown in Supplementary Table 1) was performed to optimize the hyperparameters of each algorithm.

As shown in Supplementary Table 2, the dataset was imbalanced due to the rarity of neurological deterioration (approximately 6%) among the patients with mild TBI. Thus, class weighting was applied to modify the loss function during model training to solve the problems associated with imbalanced data. Specifically, we assigned an approximately 13 times greater weight to the positive cases than to the negative cases, as reported previously (22).

### Performance evaluation

The evaluation metrics used to compare the predictive performance of the machine learning algorithms included the area under the receiver operating characteristic curve (AUROC) and the area under the precision–recall curve (AUPRC). The Youden index was used to establish the cutoff value, and the sensitivity, specificity, positive predictive value, and negative predictive value were calculated.

### Statistical analysis

R version 4.0.3 was used as the statistical analysis software. Student’s t test and Welch’s t test were applied to compare continuous and normally distributed variables between groups. For nonnormally distributed variables, the Mann–Whitney U test was used for between-group comparisons. For categorical variables, groups were compared with the chi-square test.

All tests were two-sided, and a significance level of 0.05 was used. Bonferroni adjustment was applied to mitigate the risk of Type I errors arising from performing multiple comparison tests on the same data.

## Results

**Figure 1:**
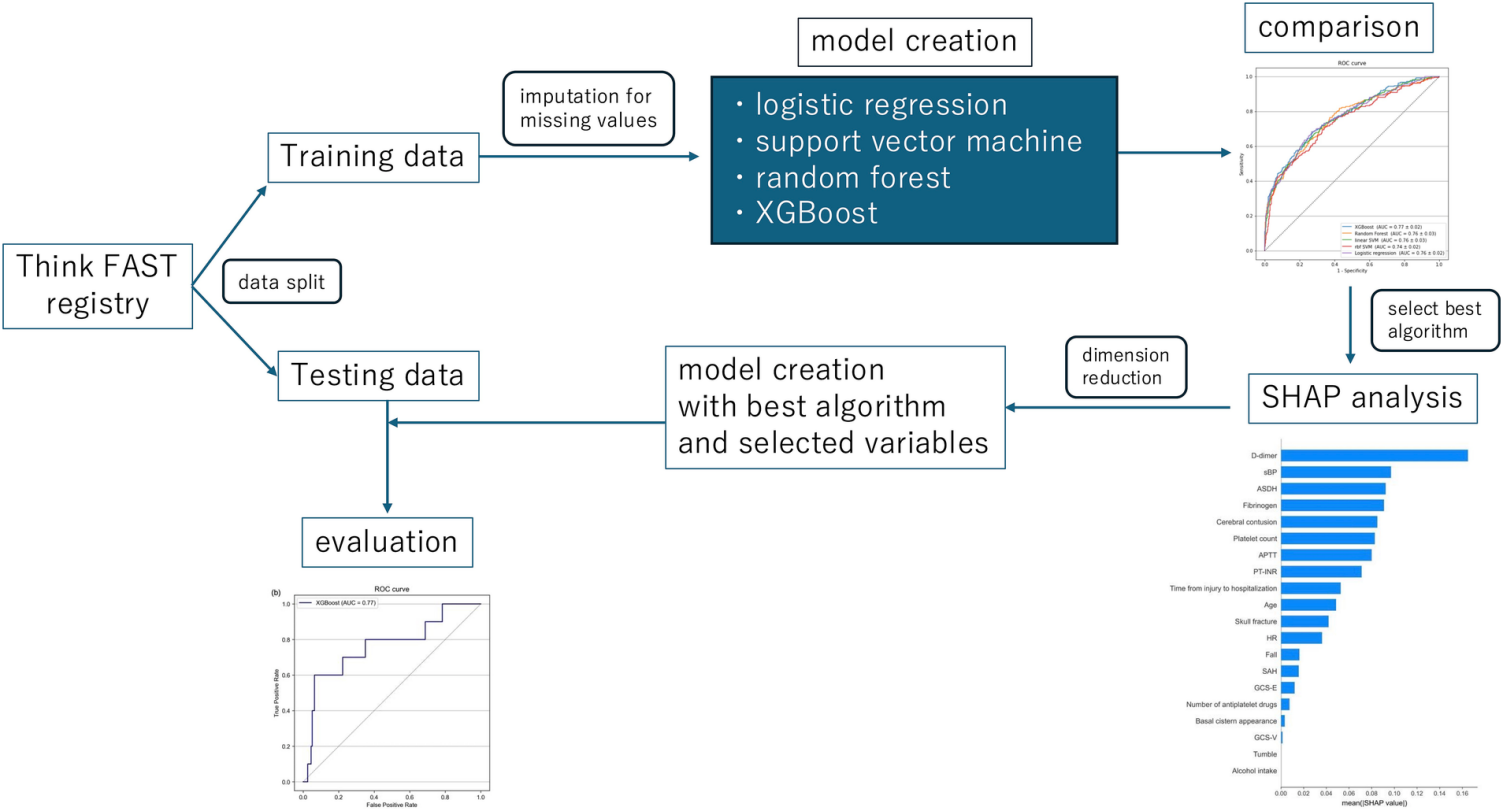
The whole study design. XGBoost, eXtreme Gradient Boosting; SHAP, the SHapley Additive exPlanations.

The Think FAST registry includes data from a total of 721 head trauma patients aged 65 years and older, among whom 421 patients had an admission GCS score of 13 points or higher and were treated with conservative management at admission (Figure 1). Neurological deterioration occurred in 25 patients (6%). The distributions of each variable in the training and validation datasets and the testing dataset are presented in Table 1.

**Table 1:** Baseline features of the study cohort.

Feature/variable	Entire cohort *N* = 417	Training and validation cohort *N* = 250	Testing cohort *N* = 167
Age, mean (SD)	79.2 (7.6)	79.1 (7.4)	79.3 (7.8)
Sex			
Men, No. (%)	244 (59)	155 (62)	89 (53)
Women, No. (%)	173 (41)	95 (38)	78 (47)
Injury mechanism			
Traffic accident, No. (%)	88 (21)	51 (20)	37 (22)
Fall, No. (%)	73 (18)	48 (19)	25 (15)
Tumble, No. (%)	236 (57)	109 (44)	95 (57)
Time from injury to hospitalization (min), median (range)	66.5 (5–10,991)	67.0 (5–8,184)	66.0 (14–10,991)
Vital signs			
sBP (mmHg), mean (SD)	150 (28)	151 (28)	151 (29)
HR (beats/min), mean (SD)	84 (16)	84 (15)	83 (17)
Glasgow Coma Scale on admission			
Eye open			
4, No. (%)	344 (82)	205 (82)	139 (83)
3, No. (%)	73 (18)	45 (18)	28 (17)
Verbal reaction			
5, No. (%)	240 (58)	139 (56)	101 (60)
4, No. (%)	167 (40)	103 (41)	64 (38)
3, No. (%)	10 (2)	8 (3)	2 (1)
Movement			
6, No. (%)	416 (99.8)	249 (99.6)	167 (100)
5, No. (%)	1 (0.2)	1 (0.4)	
Paresis			
Yes, No. (%)	14 (3)	6 (2)	8 (5)
No, No. (%)	403 (97)	244 (98)	159 (95)
Alcohol intake			
Yes, No. (%)	31 (7)	16 (6)	15 (9)
No, No. (%)	386 (93)	234 (94)	152 (91)
Antithrombotic agents			
Number of antiplatelet drugs			
Zero, No. (%)	319 (77)	183 (74)	136 (81)
One, No. (%)	83 (20)	56 (23)	27 (16)
Two, No. (%)	12 (3)	8 (3)	4 (3)
Anticoagulant drug			
Warfarin, No. (%)	22 (5)	12 (5)	10 (6)
DOAC, No. (%)	38 (9)	22 (9)	16 (10)
None, No. (%)	357 (86)	216 (86)	141 (84)
Reversal therapy for antithrombotic agents			
Vitamin K, No. (%)	14 (3)	8 (3)	6 (4)
Fresh Frozen Plasma, No. (%)	5 (1)	4 (2)	1 (1)
Four Factor Prothrombin Complex Concentrate, No. (%)	9 (2)	7 (3)	2 (1)
Idarucizumab, No. (%)	1 (0.2)	1 (0.4)	0 (0)
Platelet transfusion, No. (%)	3 (1)	2 (1)	1 (1)
Hemostatic agent			
Tranexamic acid administration			
Yes, No. (%)	119 (41)	101 (40)	71 (43)
No, No. (%)	298 (59)	149 (60)	96 (57)
Carbazochrome administration			
Yes, No. (%)	172 (29)	71 (28)	48 (29)
No, No. (%)	245 (71)	179 (72)	119 (71)
Laboratory data			
Platelet counts, mean (SD)	19.5 (7.5)	19.7 (7.9)	19.2 (6.8)
PT-INR, mean (SD)	1.17 (0.8)	1.15 (0.74)	1.20 (0.92)
APTT, mean (SD)	27.3 (5.4)	28.0 (4.3)	27.4 (6.7)
D-dimer (μg/ml), median (SD)	10.8 (47.7)	10.7 (50.3)	11.0 (41)
Fibrinogen (mg/dL)	318 (102)	321 (113)	314 (86)
Head CT findings			
ASDH			
Yes, No. (%)	208 (50)	127 (51)	81 (48)
No, No. (%)	209 (50)	123 (49)	86 (52)
Thickness in positive cases (mm), mean (SD)	5.4 (4.0)	5.3 (3.8)	5.6 (4.5)
EDH			
Yes, No. (%)	19 (5)	11 (4)	8 (5)
No, No. (%)	398 (95)	239 (96)	159 (95)
Thickness in positive cases (mm), mean (SD)	12.3 (9.1)	11.7 (7.2)	13.2 (11.7)
Cerebral contusion			
Yes, No. (%)	81 (19)	48 (19)	33 (20)
No, No. (%)	336 (81)	202 (81)	134 (80)
Diameter in positive cases (mm), mean (SD)	15.3 (14)	13.4 (10.5)	17.9 (18.1)
SAH			
Yes, No. (%)	227 (56)	136 (56)	91 (55)
No, No. (%)	190 (44)	109 (44)	73 (45)
Basal cistern appearance			
Disappear, No. (%)	2 (0.5)	0	2 (1)
Compressed, No. (%)	12 (3)	7 (3)	5 (3)
Normal, No. (%)	403 (96.5)	243 (97)	160 (96)
Midline shift			
Yes, No. (%)	23 (9)	19 (8)	10 (6)
No, No. (%)	242 (91)	231 (92)	157 (94)
Shift in positive cases (mm), mean (SD)	4.5 (2.5)	4.0 (2.4)	5.5 (2.0)
Skull fracture			
Yes, No. (%)	64 (16)	41 (17)	23 (14)
No, No. (%)	343 (84)	203 (83)	140 (86)
Skull base fracture			
Yes, No. (%)	19 (5)	16 (7)	3 (2)
No, No. (%)	389 (95)	229 (93)	160 (98)

SD, standard deviation; sBP, systolic blood pressure; HR, heart rate; DOAC, direct oral anticoagulant; PT-INR, Prothrombin Time-International Normalized Ratio; APTT, Activated Partial Thromboplastin Time; ASDH, acute subdural hematoma; EDH, epidural hematoma; SAH, subarachnoid hemorrhage.

The distribution of missing values is illustrated in Supplementary Figure 1. High rates of missing values were observed, particularly in the coagulation profiles, notably the D-dimer and fibrinogen levels. Little’s MCAR test revealed a *p* value of 0.93, indicating that none of the missing values were MCAR. Missing values were imputed via k-nearest neighbor, multiple imputation, and random forest regression methods.

All the data were randomly divided into a training/validation dataset and a testing dataset such that the proportion of data corresponding to patients who experienced neurological deterioration remained consistent across both datasets. Sixty percent of the data (those in the training/validation dataset) were utilized to construct the predictive model through cross-validation, whereas the remaining 40% (testing dataset) were used to test the performance of the constructed predictive models.

**Figure 2:**
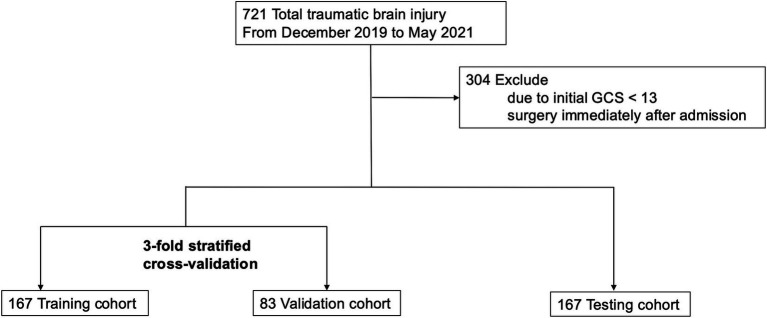
Study flow chart.

Initially, predictive models were created using all available data extracted from the Think FAST registry. XGBoost, random forest, support vector machine (with a linear RBF kernel), and logistic regression frameworks were employed to construct the predictive models. XGBoost, in particular, is designed to allow the construction of predictive models without the need to impute missing values. We then compared the performance of each machine learning model and found that the model constructed using XGBoost without imputing missing values had the highest predictive performance, with an AUROC of 0.81 [0.07] and an AUPRC of 0.33 [0.08] (Table 2 and Supplementary Table 3). The mean absolute SHAP values were also calculated and are presented in Figure 2.

**Table 2:** Area under the receiver operating characteristics curve in each algorithm with each imputation method.

Algorithm	k-nn	Multiple imputation	Regression imputation	Without imputation
XGBoost, mean (SD)	0.73 (0.03)	0.78 (0.07)	0.78 (0.09)	0.81 (0.07)
Random Forest, mean (SD)	0.67 (0.12)	0.71 (0.10)	0.75 (0.07)	N/A
Linear SVM, mean (SD)	0.61 (0.11)	0.61 (0.12)	0.61 (0.12)	N/A
RBF SVM, mean (SD)	0.71 (0.04)	0.69 (0.04)	0.69 (0.04)	N/A
Logistic Regression, mean (SD)	0.65 (0.12)	0.65 (0.13)	0.65 (0.13)	N/A

SD, standard deviation; N/A, not applicable; RBF, radial basis function; k-nn, k-nearest neighbors.

**Figure 3:**
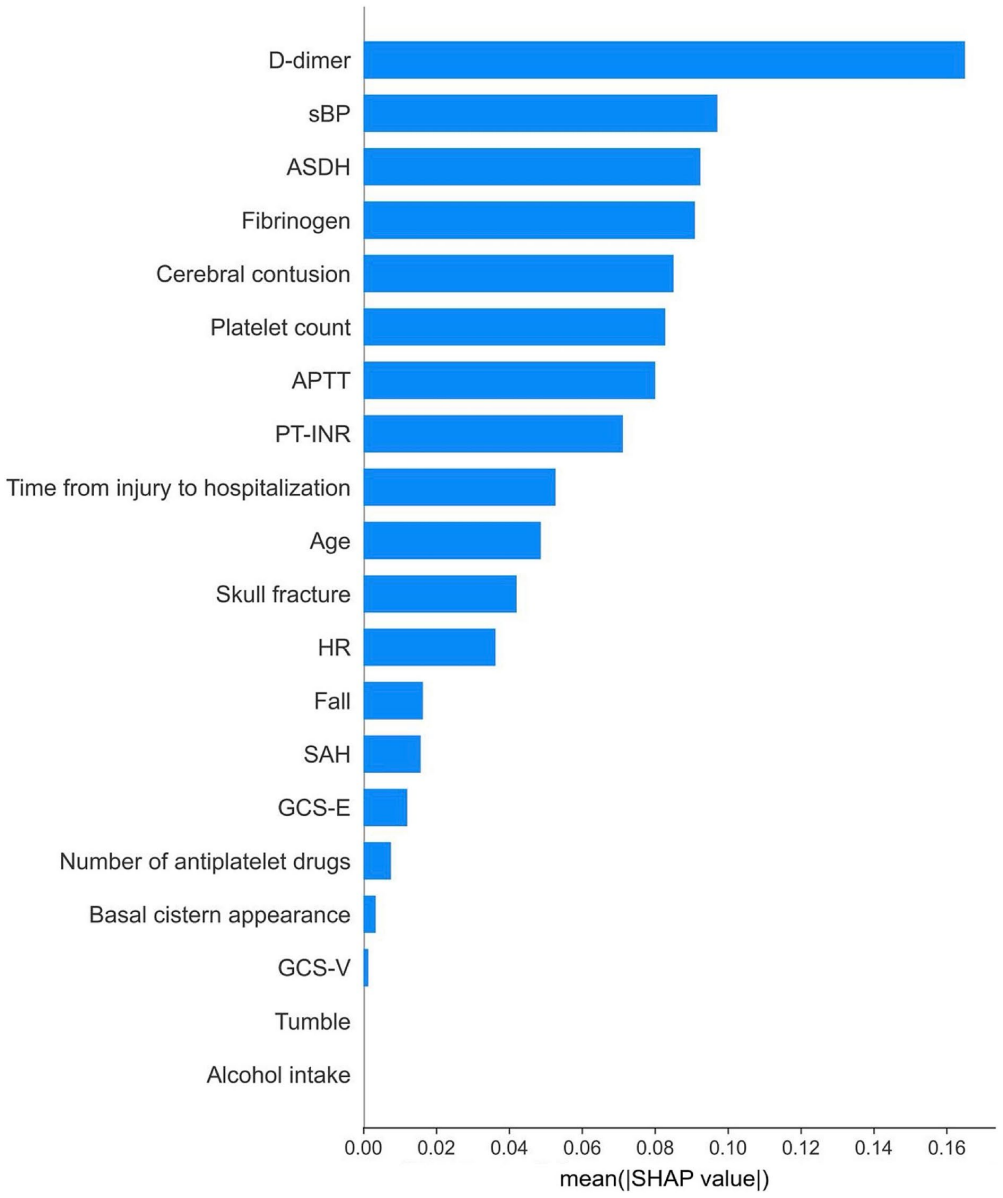
Relative importance of features according to their absolute SHAP values.

**Figure 4:**
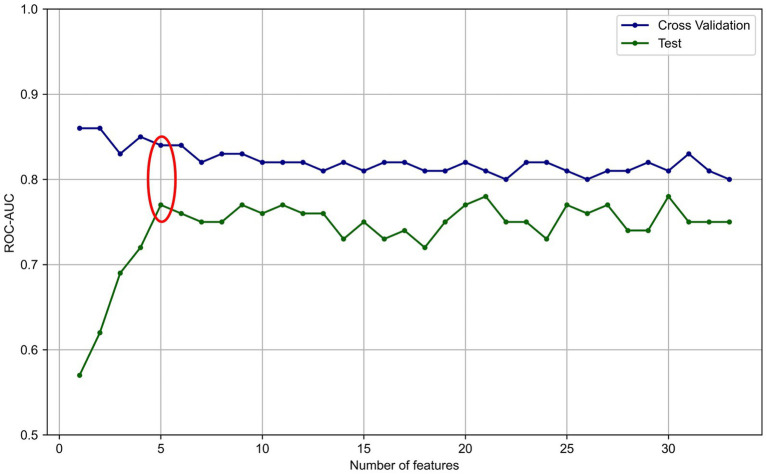
The AUROC trends in the training/validation dataset and testing dataset after dimension reduction based on SHAP values. The red circle indicates the results of feature numbers that showed superior AUROC in cross-validation and test. AUROC, area under the receiver operating characteristic curve.

Next, variables with low mean absolute SHAP values were sequentially removed for dimensionality reduction. The AUROC trends in the training/validation dataset and testing dataset after reconstruction of the XGBoost model with the different sets of dimensionally reduced features are shown in Figure 3. Among the various models, the predictive model constructed using the top five variables identified through SHAP analysis demonstrated the highest AUROC during cross-validation and showed minimal differences in predictive accuracy in the testing data. For this model, the cross-validation AUROC was 0.84, and the AUPRC was 0.34, whereas in the testing dataset, the AUROC was 0.77, and the AUPRC was 0.19 (Figure 4 and Supplementary Figure 2). Using the bootstrap method, the 95% confidence interval for the AUROC in the testing dataset was calculated to be 0.57–0.94. This range indicates a significantly higher predictive accuracy of this model than that of random guessing.

**Figure 5:**
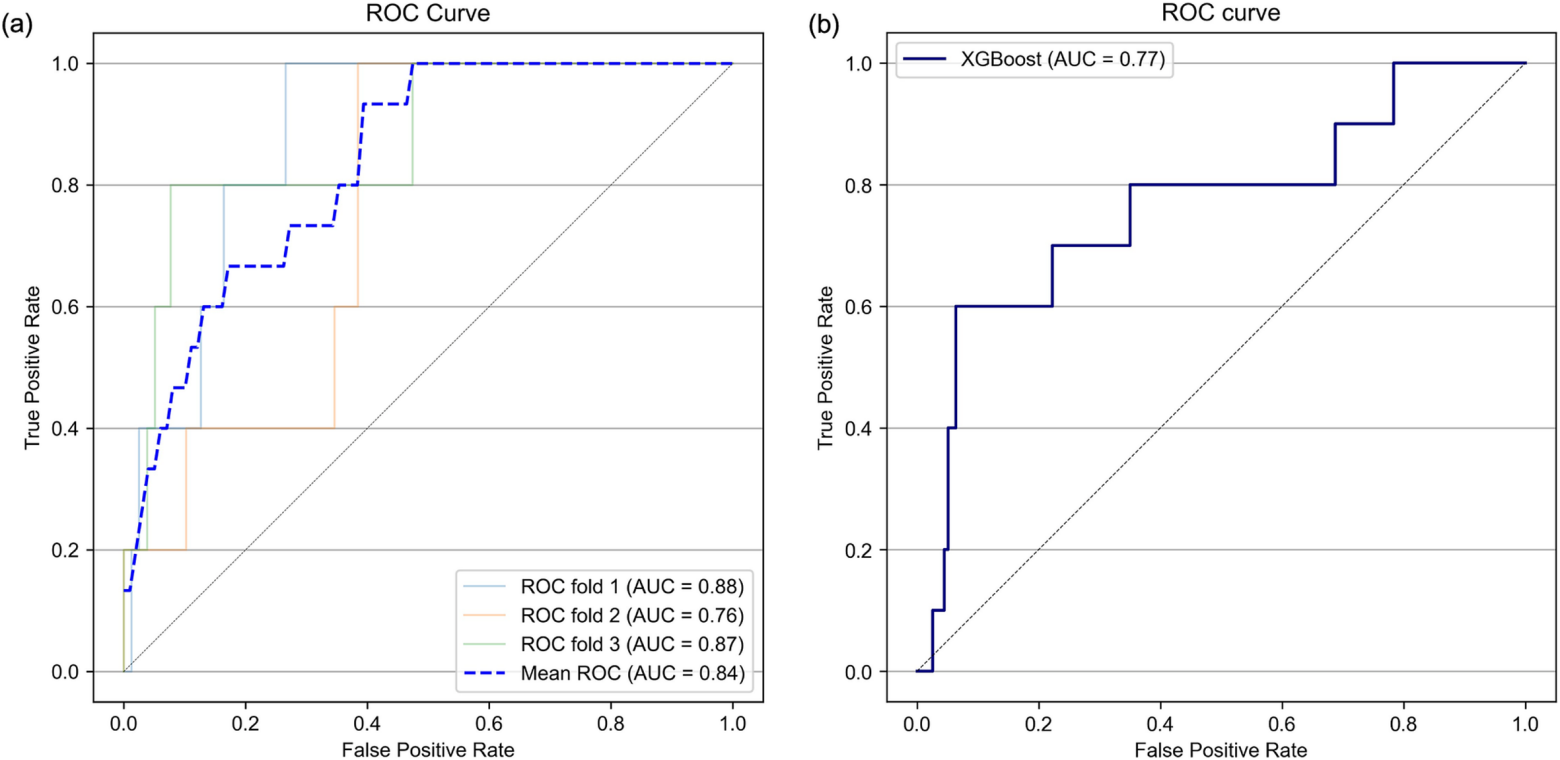
Receiver operating characteristic (ROC) curves in the threefold stratified cross-validation **(A)** and in the testing dataset **(B)**. AUC, area under the curve.

**Figure 6:**
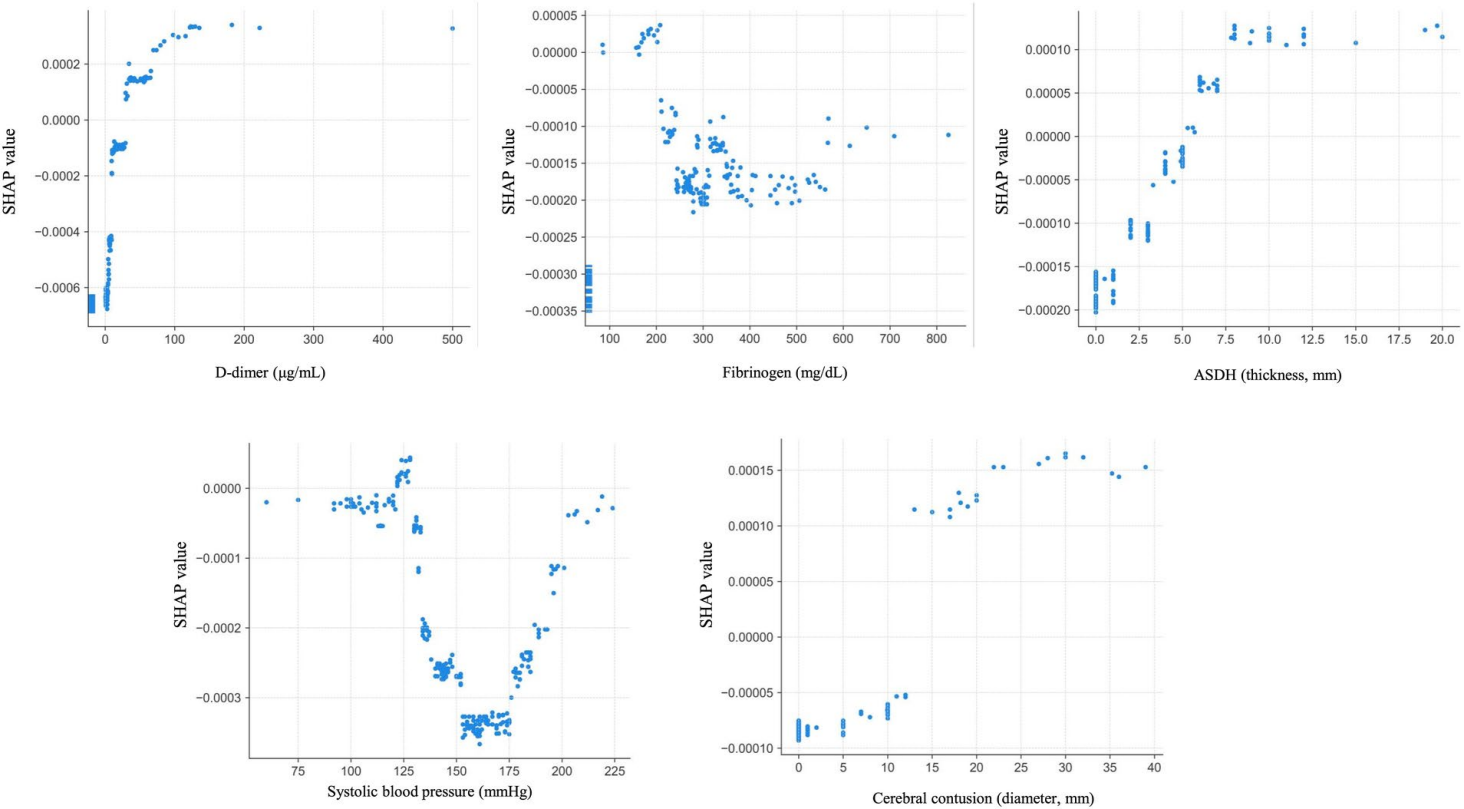
Dependence plots for the five most important features. ASDH: acute subdural hematoma.

The SHAP dependence plot, which illustrates the distribution between the numerical values of each variable and their corresponding SHAP values in the prediction model, is presented in Figure 5. For D-dimer levels, a transition in SHAP values from negative to positive occurred when the level surpassed 30 μg/mL, indicating a tendency for the SHAP value to increase as the D-dimer level increased. Fibrinogen levels displayed a shift toward positive SHAP values when at levels below 200 mg/dL, at which they contributed more to neurological deterioration. For the acute subdural hematoma (ASDH) thickness, a transition from negative to positive SHAP values occurred at a thickness of 5 mm, whereas a cerebral contusion exceeding 10 mm in size showed a similar trend toward neurological deterioration (Figure 6).

At the cutoff value for this model determined via the Youden index, the sensitivity was 60%, the specificity was 96%, the positive predictive value was 37.5%, and the negative predictive value was 90%. When three neurosurgeons reviewed the data for each variable in the test dataset to predict the occurrence of neurological deterioration, the sensitivity was 20%, the specificity was 94%, the positive predictive value was 19%, and the negative predictive value was 94% (Table 3).

**Table 3:** Comparison of neurological deterioration as assessed by the XGBoost model and by neurosurgeons.

Predictor	Sensitivity	Specificity	PPV	NPV
XGBoost	60	96	38	90
Neurosurgeons, mean (SD)	20 (0)	94 (1.0)	19 (1.0)	94 (0.4)

SD, standard deviation; PPV, positive predictive value; NPV, negative predictive value.

## Discussion

In this study, we constructed a machine learning model to predict neurological deterioration among patients with mild TBI via data from the Think FAST registry database, which comprises data prospectively collected from multiple facilities, particularly from patients aged 65 years and older. Among several machine learning models constructed with one of several imputation methods, the model constructed via the XGBoost framework without imputation showed comparatively better predictive performance than the other models did, with an AUROC of 0.81 according to cross-validation. Furthermore, by conducting feature dimension reduction on the basis of the feature SHAP values, we built a predictive model using XGBoost that could predict neurological deterioration with an AUROC of 0.84 in cross-validation and an AUROC of 0.77 in the testing dataset.

Neurological deterioration after mild TBI has long been recognized; patients with this condition are said to “talk and deteriorate” or “talk and die” (23–25). Marshal et al.’s study published in 1984 (26) reported a TBI incidence of approximately 10% in head trauma patients, with over half of them dying or being left comatose. According to the report by Lobato et al. (27), among patients with severe TBI, approximately 25% were able to converse before deteriorating to a severe state, with 32% of these patients ultimately dying. Therefore, neurological deterioration has been acknowledged as a condition that emerges in some patients with TBI and results in a poor prognosis, posing a clinical challenge to differentiate patients who experience neurological deterioration from those who do not. Recent meta-analyses have indicated that neurological deterioration occurs in approximately 12% of patients with mild TBI; however, statistically significant prognostic factors for neurological deterioration have not been identified (28). Moreover, to our knowledge, methods for stratifying the risk of neurological deterioration remain elusive. In this study, the best predictive model we constructed achieved good predictive accuracy, with an AUROC of 0.77 for predicting neurological deterioration in the testing dataset. This performance indicates a predictive accuracy superior to that of random guessing and suggests the potential for better sensitivity and a greater positive predictive value than predictions made by neurosurgeons.

For this predictive model, we extracted data on variables that are highly correlated with neurological deterioration via SHAP analysis. The D-dimer level, fibrinogen level, ASDH thickness, systolic blood pressure, and cerebral contusion diameter were selected as the most important variables. The trends in the SHAP values for these variables in this study were consistent with the results of previous reports on the severity of TBI and neurological deterioration. For example, regarding the relationship between coagulation parameters such as D-dimer and fibrinogen levels and TBI, Nakae et al. reported that the D-dimer level tends to increase while the fibrinogen level tends to decrease early after injury (29). Moreover, patients with TBI and elevated D-dimer levels upon admission are more prone to hemorrhage progression and neurological deterioration, resulting in poorer outcomes for patients with elevated D-dimer levels than those without elevated D-dimer levels (30, 31). Head CT studies have revealed that ASDH thickness is a risk factor for subsequent exacerbation (32), and cerebral contusions measuring larger than 20 mm are significantly associated with hemorrhage progression (33). These trends align with the dependence plots shown for each variable, indicating the consistency of these results. In this study, we not only focused on these individual factors but also analyzed their complex interrelationships via machine learning techniques to construct a prediction model to accurately predict neurological deterioration.

The number of studies using machine learning in the field of medicine has rapidly increased in recent years (34). In particular, predictive models that utilize ensemble learning or deep learning have been shown to demonstrate superior predictive accuracy to traditional logistic regression models in many studies (35). However, the black-box nature of the prediction process inherent in these complex models poses a barrier to their practical application in clinical settings. SHAP analysis is a method that was developed for interpreting machine learning models (36), allowing interpretation of the meaning of the features used in the construction of predictive models. In this study, by visualizing the trends of feature importance via dependence plots, the information obtained from the analysis could be used to increase the reliability of the machine learning model. The predictive model developed in this study is publicly available on the GitHub website (37) in a format that can be easily implemented. By utilizing our model, it may be possible to detect cases of deterioration at an early stage after hospitalization. This could contribute to improving the prognosis of TBI by implementing strict monitoring and early follow-up for high-risk patients. Furthermore, enhancing predictive models, such as the one in this study, is expected to advance personalized medicine for patients with TBI.

### Limitations

As the Think FAST registry used in this study contains data from multiple facilities collected in a prospective manner, the impact of domain shifts is considered relatively minimal. Although the predictive model we developed did not show a significant discrepancy in AUROC between cross-validation and testing, the overall sample size was too small to establish an external validation dataset. Future external validation studies will be necessary to confirm the validity of this model. In addition, since the Think FAST registry includes data from patients aged 65 years and older, our predictive model can only be applied to older patients. Since the mechanisms of injury, types of hemorrhage, and clinical courses of TBI may differ between younger and older patients, applying our present model to populations with different prior probabilities could yield incorrect results. Therefore, for younger patients, it is necessary to develop another predictive model or at least validate the present model using data with a wider age range.

Furthermore, the variables used to create this model included only those for which data, such as clinical examination data, laboratory data and descriptive findings from head CT scans, are commonly acquired in the management of head trauma. In the future, CT images could be incorporated into predictive models via convolutional neural networks to increase the predictive accuracy of these models. Additionally, since this study focused solely on the progression of consciousness impairment after admission as the outcome, further investigation is needed to understand how this information could be utilized in treatment decision-making and its impact on patient outcomes.

## Conclusion

The application of machine learning models suggests the potential to detect the occurrence of neurological deterioration in elderly patients with mild TBI using only admission data. Further validation with an external dataset is needed in the future.

## Data Availability

The data analyzed in this study is subject to the following licenses/restrictions: data sharing requires the permission by Think FAST registry group. Requests to access these datasets should be directed to inamnsrg@tmd.ac.jp.
