# Supplementary figures and images for: A machine learning model to predict neurological deterioration after mild traumatic brain injury in older adults
Source: Front Neurol. 2025 Jan 3;15:1502153. doi: 10.3389/fneur.2024.1502153 (PMC11739101; doi:10.3389/fneur.2024.1502153)

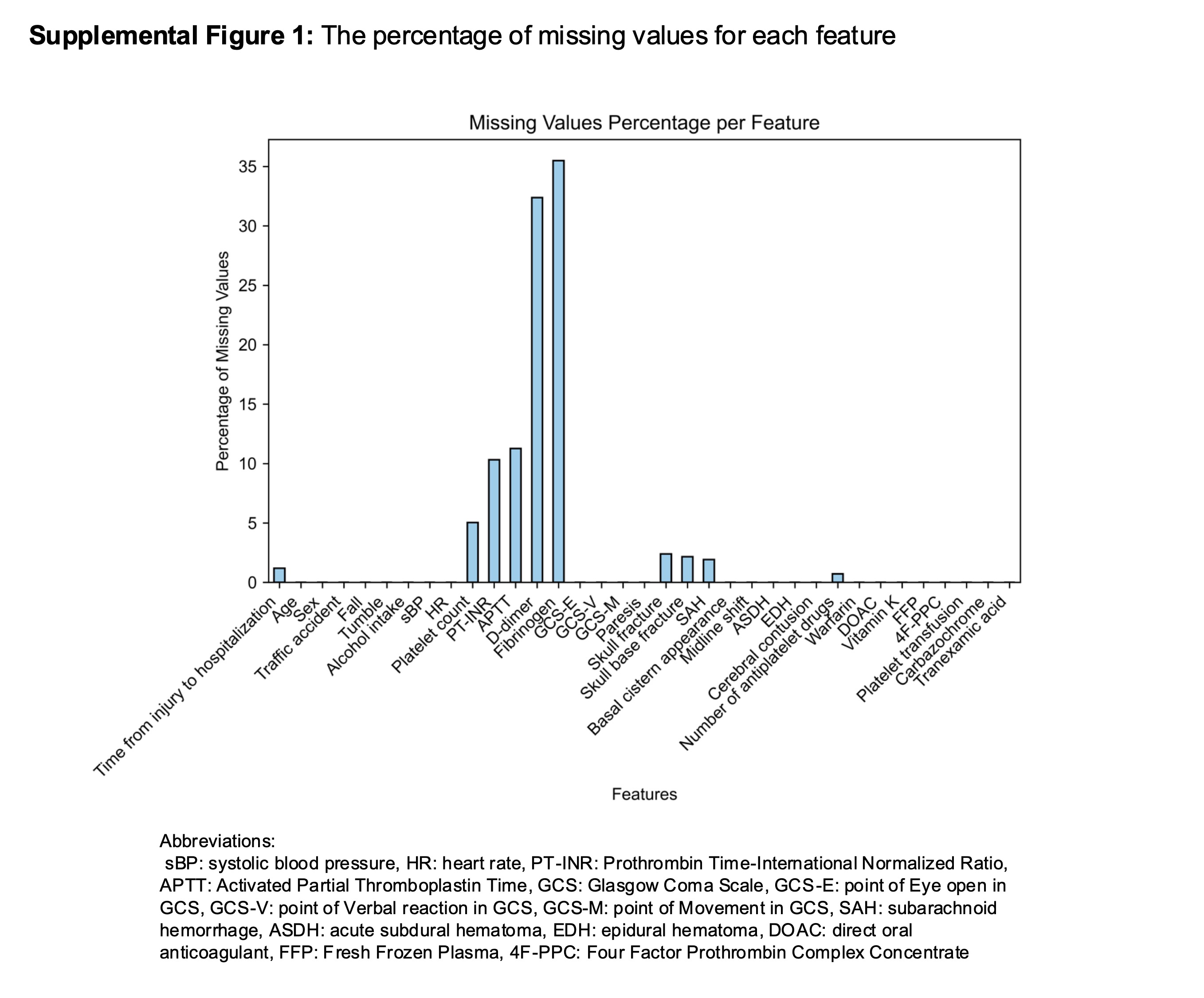

Supplement: Supplementary file 1 [file Image_1.JPEG]

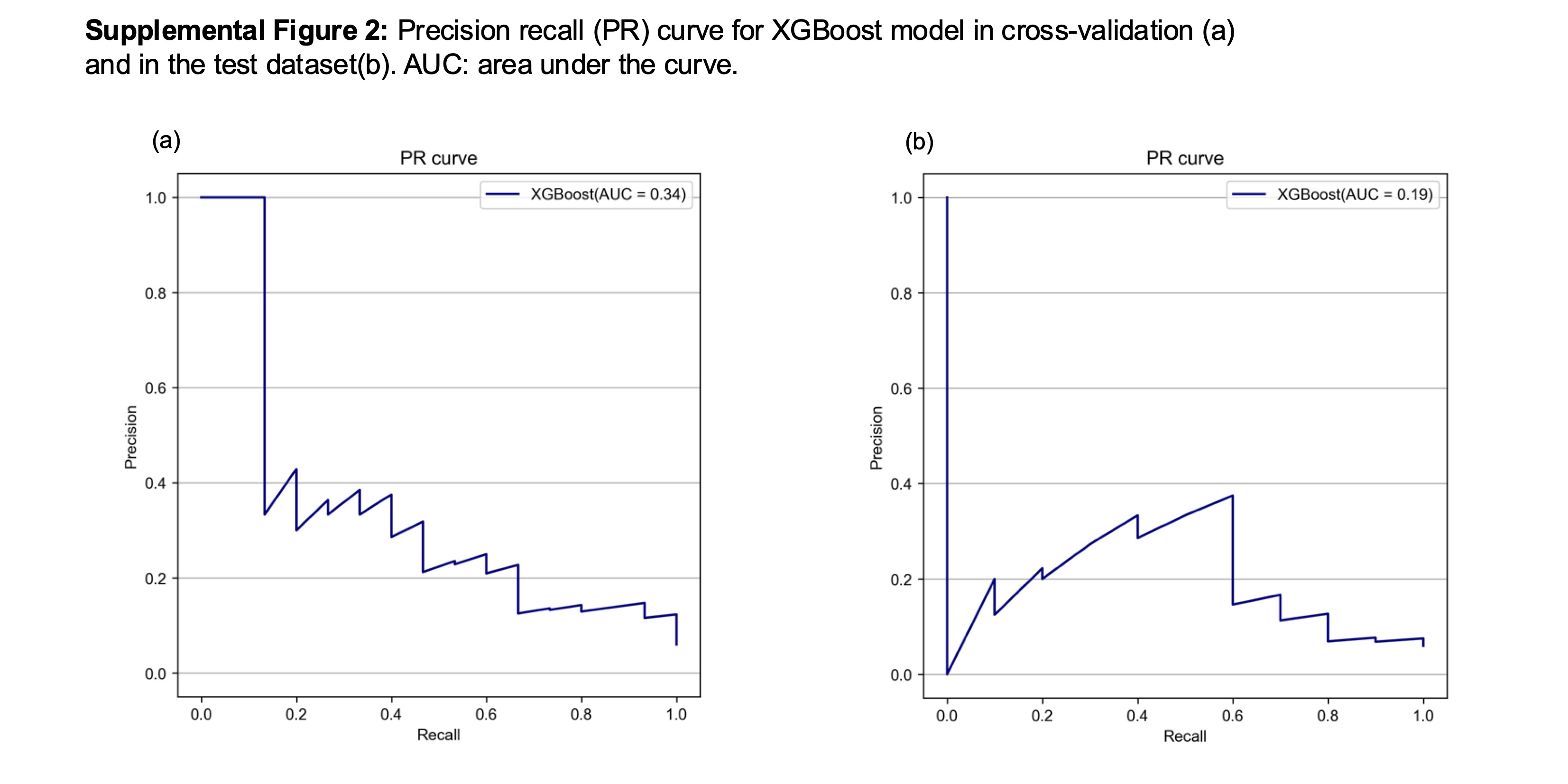

Supplement: Supplementary file 2 [file Image_2.JPEG]
