# Supplementary material for: A machine learning model to predict neurological deterioration after mild traumatic brain injury in older adults
Source: Front Neurol. 2025 Jan 3;15:1502153. doi: 10.3389/fneur.2024.1502153 (PMC11739101; doi:10.3389/fneur.2024.1502153)
Supplement: Supplementary file 3 [file Table_1.DOCX]

**Supplemental Table 1:** The values of hyper parameters for each algorithm

| Algorithm /  hyper parameter | the values of hyper parameters |
| --- | --- |
| XGBoost |  |
| learning_rate | (0.0001, 0.001, 0.01, 0.1, 0.2, 0.3) |
| max_depth | (2, 4, 6, 8, 10, 12) |
| subsample | (0.2, 0.4, 0.6, 0.8, 1.0) |
| colsample_bytree | (0.2, 0.4, 0.6, 0.8, 1.0) |
| min_child_weight | (2, 4, 6, 8) |
| Random Forest |  |
| criterion | (“gini”, “entropy”) |
| n_estimators | (40, 60, 80, 100, 200) |
| max_features | ((1, 2, 3), “auto”) |
| max_depth | (2, 4, 6, 8, 10, 20, “None”) |
| min_samples_leaf | (1, 2, 3, 4, 5) |
| SVM |  |
| kernel | (“linear”, “rbf”) |
| C | (0.01, 0.1, 1, 10, 100) |
| gamma | (0.01, 0.1, 1, 10, 100) |
| Logistic regression |  |
| C | (0.001, 0.005, 0.01, 0.02, 0.03, 0.05, 0.1, 0.2, 0.3, 0.5, 1, 5, 10, 100) |
| max_iter | (50, 100, 300, 500, 1000) |
| penalty | (“l1”, “l2”) |
| solver | (“newton-cg”, “lbfgs”, “liblinear”, “sag”, “saga”) |
