# Supplementary material for: A machine learning model to predict neurological deterioration after mild traumatic brain injury in older adults
Source: Front Neurol. 2025 Jan 3;15:1502153. doi: 10.3389/fneur.2024.1502153 (PMC11739101; doi:10.3389/fneur.2024.1502153)
Supplement: Supplementary file 4 [file Table_2.DOCX]

**Supplemental Table 2:** Characteristics of the patients in the study group

| Feature/Variable | Entire cohort N=417 | Clinical deterioration (-) N=392 | Clinica deterioration (+) N=25 |
| --- | --- | --- | --- |
| Age, mean (SD) | 79.2 (7.6) | 79.0 (7.6) | 80.2 (8.2) |
| Sex |  |  |  |
| Men, No. (%) | 244 (59) | 229 (58) | 15 (60) |
| Women, No. (%) | 173 (41) | 163 (42) | 10 (40) |
| Injury Mechanism |  |  |  |
| Traffic accident, No. (%) | 88 (21) | 85 (22) | 3 (12) |
| Fall, No. (%) | 73 (18) | 67 (17) | 6 (24) |
| Tumble, No. (%) | 236 (57) | 223 (57) | 12 (48) |
| Time from injury to hospitalization(min), median (range) | 66.5 (5 - 10991) | 68.0 (5 - 10091) | 56.0 (5 - 724) |
| Vital signs |  |  |  |
| sBP(mmHg), mean (SD) | 150 (28) | 151 (28) | 148 (30) |
| HR (beats/min), mean (SD) | 84 (16) | 84 (16) | 83 (17) |
| Glasgow Coma Scale on admission |  |  |  |
| Eye open |  |  |  |
| 4, No. (%) | 344 (82) | 323 (82) | 21 (84) |
| 3, No. (%) | 73 (18) | 69 (18) | 4 (16) |
| Verbal reaction |  |  |  |
| 5, No. (%) | 240 (58) | 228 (58) | 12 (48) |
| 4, No. (%) | 167 (40) | 155 (40) | 12 (48) |
| 3, No. (%) | 10 (2) | 9 (2) | 1 (4) |
| Movement |  |  |  |
| 6, No. (%) | 416 (99.8) | 391 (99.7) | 25 (100) |
| 5, No. (%) | 1 (0.2) | 1 (0.3) | 0 |
| Paresis |  |  |  |
| yes, No. (%) | 14 (3) | 12 (3) | 2 (8) |
| no, No. (%) | 403 (97) | 380 (97) | 23 (92) |
| Alcohol intake |  |  |  |
| yes, No. (%) | 31 (7) | 363 (7) | 2 (8) |
| no, No. (%) | 386 (93) | 29 (93) | 23 (92) |
| Antithrombotic agents |  |  |  |
| Number of antiplatelet drugs |  |  |  |
| zero, No. (%) | 319 (77) | 301 (77) | 18 (72) |
| one, No. (%) | 83 (20) | 77 (20) | 6 (24) |
| two, No. (%) | 12 (3) | 11 (3) | 1 (4) |
| Anticoagulant drug |  |  |  |
| warfarin, No. (%) | 22 (5) | 22 (6) | 0 |
| DOAC, No. (%) | 38 (9) | 36 (9) | 2 (8) |
| none, No. (%) | 357 (86) | 334 (85) | 23 (92) |
| Reversal therapy for antithrombotic agents |  |  |  |
| Vitamin K, No. (%) | 14 (3) | 14 (4) | 0 |
| Fresh Frozen Plasma, No. (%) | 5 (1) | 4 (1) | 1 (4) |
| Four Factor Prothrombin Complex Concentrate, No. (%) | 9 (2) | 9 (2) | 0 |
| Idarucizumab, No. (%) | 1 (0.2) | 1 (0.3) | 0 |
| Platelet transfusion, No. (%) | 3 (1) | 3 (1) | 0 |
| Hemostatic agent |  |  |  |
| Tranexamic acid administration |  |  |  |
| yes, No. (%) | 119 (41) | 165 (58) | 7 (28) |
| no, No. (%) | 298 (59) | 227 (42) | 18 (72) |
| Carbazochrome administration |  |  |  |
| yes, No. (%) | 172 (29) | 117 (30) | 2 (8) |
| no, No. (%) | 245 (71) | 275 (70) | 23 (92) |
| Laboratory data |  |  |  |
| platelet counts, mean (SD) | 19.5 (7.5) | 19.3 (7.6) | 16.9 (5.2) |
| PT-INR, mean (SD) | 1.17 (0.80) | 1.18 (0.80) | 1.07 (0.13) |
| APTT, mean (SD) | 27.3 (5.4) | 27.4 (5.5) | 26.7 (2.8) |
| D-dimer (μg/ml), median (SD) | 10.75 (46.7) | 10.3 (44.4) | 34.2 (61.2) |
| Fibrinogen (mg/dL), median (SD) | 297 (102) | 297 (102) | 286 (102) |
| Head CT findings |  |  |  |
| ASDH |  |  |  |
| yes, No. (%) | 208 (50) | 192 (49) | 16 (64) |
| no, No. (%) | 209 (50) | 200 (51) | 9 (36) |
| thickness in positive cases (mm), mean (SD) | 5.4 (4.0) | 5.3 (4.1) | 6.2 (2.5) |
| EDH |  |  |  |
| yes, No. (%) | 19 (5) | 17 (4) | 2 (8) |
| no, No. (%) | 398 (95) | 375 (96) | 23 (92) |
| thickness in positive cases (mm), mean (SD) | 12.3 (9.1) | 13.0 (9.4) | 7.0 (4.2) |
| cerebral contusion |  |  |  |
| yes, No. (%) | 81 (19) | 72 (18) | 9 (36) |
| no, No. (%) | 336 (81) | 320 (82) | 16 (64) |
| diameter in positive cases (mm), mean (SD) | 15.3 (14) | 14.1 (11.6) | 24.8 (26.6) |
| SAH |  |  |  |
| yes, No. (%) | 227 (56) | 210 (55) | 17 (68) |
| no, No. (%) | 182 (44) | 174 (45) | 8 (32) |
| basal cistern appearance |  |  |  |
| disappear, No. (%) | 2 (0.5) | 2 (1) | 0 |
| compressed, No. (%) | 12 (3) | 9 (2) | 3 (12) |
| normal, No. (%) | 403 (96.5) | 381 (97) | 22 (88) |
| midline shift |  |  |  |
| yes, No. (%) | 29(7) | 26 (7) | 3 (12) |
| no, No. (%) | 388 (93) | 366 (93) | 22 (88) |
| shift in positive cases (mm), mean (SD) | 4.5 (2.4) | 4.4 (2.3) | 5.3 (3.1) |
| skull fracture |  |  |  |
| yes, No. (%) | 64 (16) | 57 (15) | 7 (28) |
| no, No. (%) | 343 (84) | 325 (85) | 18 (72) |
| skull base fracture |  |  |  |
| yes, No. (%) | 19 (5) | 19 (5) | 0 |
| no, No. (%) | 389 (95) | 364 (95) | 25 (100) |

Abbreviations:

SD: standard deviation, sBP: systolic blood pressure, HR: heart rate, DOAC: direct oral anticoagulant, PT-INR: Prothrombin Time-International Normalized Ratio, APTT: Activated Partial Thromboplastin Time, ASDH: acute subdural hematoma, EDH: epidural hematoma, SAH: subarachnoid hemorrhage
