# Supplementary material for: A machine learning model to predict neurological deterioration after mild traumatic brain injury in older adults
Source: Front Neurol. 2025 Jan 3;15:1502153. doi: 10.3389/fneur.2024.1502153 (PMC11739101; doi:10.3389/fneur.2024.1502153)
Supplement: Supplementary file 5 [file Table_3.DOCX]

**Supplemental Table 3:** Area under the Precision Recall curve in each algorithm with each imputation method

| Algorithm | k-nn | multiple imputation | regression imputation | without imputation |
| --- | --- | --- | --- | --- |
| XGBoost, mean (SD) | 0.22 (0.06) | 0.32 (0.14) | 0.30 (0.10) | 0.33 (0.08) |
| Random Forest, mean (SD) | 0.29 (0.21) | 0.28 (0.21) | 0.34 (0.22) | N/A |
| linear SVM, mean (SD) | 0.16 (0.08) | 0.16 (0.08) | 0.16 (0.08) | N/A |
| rbf SVM, mean (SD) | 0.22 (0.02) | 0.16 (0.02) | 0.16 (0.02) | N/A |
| Logistic Regression, mean (SD) | 0.20 (0.07) | 0.20 (0.07) | 0.20 (0.07) | N/A |

Abbreviations

SD: standard deviation, N/A: not applicable, rbf: radial basis function, k-nn: k-nearest neighbors
